# The cyclophilin inhibitor CRV431 inhibits liver HBV DNA and HBsAg in transgenic mice

**DOI:** 10.1371/journal.pone.0217433

**Published:** 2019-06-10

**Authors:** Philippe Gallay, Daren Ure, Michael Bobardt, Udayan Chatterji, James Ou, Daniel Trepanier, Robert Foster

**Affiliations:** 1 Department of Immunology & Microbiology, The Scripps Research Institute, La Jolla, California, United States of America; 2 ContraVir Pharmaceuticals Inc., Edison, New Jersey, United States of America; 3 Department of Molecular Microbiology and Immunology, University of Southern California, Keck School of Medicine, Los Angeles, California, United States of America; Indiana University, UNITED STATES

## Abstract

Hepatitis B virus (HBV) infection is a major health burden worldwide with 240 million chronically infected individuals. Nucleos(t)ide analogs and interferons are the current standards of care due to their suppression of HBV replication, but the treatments rarely eradicate HBV from individuals. Similar to current treatments for human immunodeficiency virus type-1 (HIV-1) and hepatitis C virus (HCV) patients, improved HBV therapies will require the combination of multiple drugs which target distinct steps of the HBV life cycle. In this study, we tested the potential of a cyclophilin inhibitor, CRV431, to affect HBV replication in transgenic mice. We found that oral treatment with CRV431 (50 mg/kg/day) for a period of 16 days significantly reduced liver HBV DNA levels and moderately decreased serum HBsAg levels. We observed an additive inhibitory effect on liver HBV DNA levels in mice treated with a combination of low doses of CRV431 (10 mg/kg/day) and the nucleotide prodrug, tenofovir exalidex (TXL), (5 mg/kg/day). No toxicity was observed in CRV431-treated mice. Although it is well known that CRV431 neutralizes the peptidyl-prolyl isomerase activity of cyclophilins, its anti-HBV mechanism(s) of action remains unknown. Nevertheless, this study provides the first demonstration of a beneficial effect of a cyclophilin inhibitor *in vivo* in an HBV transgenic mouse model. Altogether our data reveal the potential of CRV431 to be part of improved new therapies for HBV patients.

## Introduction

Hepatitis B virus (HBV) infection is a major health burden worldwide with approximately 240 million chronically infected individuals [1,2]. Chronic HBV infection increases the risk of developing liver diseases such as fibrosis, cirrhosis, and hepatocellular carcinoma [3–5]. Current therapies include interferons (IFN)s and nucleos(t)ide analogs [6–8]. IFN alpha and pegylated IFN alpha (PegIFN alpha) enhance the host immune response and block HBV replication. The nucleos(t)ide analogs adefovir, entecavir, lamivudine, telbivudine and tenofovir prevent HBV reverse transcription and replication, leading to a beneficial impact on the development of viral pathogenesis. Nevertheless, nucleos(t)ide analogs fail to completely eradicate HBV from infected cells due to the resiliency of the HBV genome, which forms a stable minichromosome—the covalently closed circular DNA (cccDNA)—in the nucleus of hepatocytes. A cure for HBV will likely require the elimination of cccDNA from infected hepatocytes. Reminiscent of current treatments for human immunodeficiency virus type-1 (HIV-1) and hepatitis C virus (HCV) patients [9,10], improved HBV therapies will require the combination of multiple drugs which target distinct steps of the HBV life cycle.

Cyclophilin inhibitors have been shown to hamper the replication of diverse viruses including HIV-1, HCV and more recently nidoviruses (arteriviruses and coronaviruses) [11–13]. Their most striking inhibitory effect was demonstrated for HCV [14–20]. Specifically, the cyclophilin inhibitor alisporivir/Debio-025 exhibited high antiviral potency *in vitro* as well as in HCV-infected patients in phase I, II, and III studies [14–21]. There are two structurally distinct main classes of non-immunosuppressive cyclophilin inhibitors: i) the cyclosporine A (CsA) analogs such as alisporivir, CRV431 (previously named CPI-431-32), SCY-635, NIM811 and STG-175; and ii) the sangliferin analogs such as NV556 (previously named BC556/NVP018) [12, 22]. Both classes of cyclophilin inhibitors neutralize the peptidyl-prolyl *cis-trans* isomerase (“foldase”) activity of members of the cyclophilin family by binding to their enzymatic hydrophobic pockets [12, 22]. Both classes of cyclophilin inhibitors show efficacy against HIV-1 and HCV [12, 22] because they block the formation of complexes between cyclophilins—especially the abundant cytosolic cyclophilin A (CypA)—and the respective viral ligands, HIV-1 capsid [23–25] and HCV NS5A [26–29]. It has been postulated that the inhibitors disrupt the proper folding of HIV-1 capsid and HCV NS5A and in turn the optimal progression of the viruses through their life cycles and productive infection of CD4+ cells and hepatocytes, respectively.

Recent studies suggest that cyclophilin inhibitors may also reduce HBV infection. Two independent studies from the Urban and Wakita laboratories showed that CsA and non-immunosuppressive CsA analogs inhibit HBV infection *in vitro* by blocking viral entry by binding to the membrane transporter sodium taurocholate co-transporting polypeptide (NTCP) with or without interfering with its transporter activity [30–32]. Other studies suggested an inhibition of HBV infection by cyclophilin inhibitors separate from their NTCP-mediated viral entry block activity [33–34]. One study showed that the CsA analog alisporivir reduced intracellular and secreted HBV DNA in HepG2.2.15, Huh7, and HepaRG cells without affecting viral entry [33]. Knockdown of CypA, CypC or CypD reduced levels of HBV DNA and intracellular of HBsAg levels, suggesting that cyclophilin members facilitate HBV replication [33]. Another study showed that the sanglifehrin analog NV556 (formerly called BC556/NVP018), which should not bind to NTCP due to its chemical structure completely dissimilar from CsA analogs [22], inhibits viral replication in NTCP-negative HepG2 cells stably expressing replicating HBV [34]. Together these data suggest that cyclophilin inhibitors may inhibit HBV infection and replication at two distinct steps—entry and post-entry.

To further determine whether cyclophilin inhibitors may represent a novel class of anti-HBV agents *in vivo*, we tested their inhibitory effect in HBV transgenic mice carrying a complete copy of the viral genome. We have found that the non-immunosuppressive CsA analog, CVRV431, profoundly reduced liver HBV DNA levels and moderately reduced serum HBsAg levels in HBV transgenic mice. In contrast, CRV431 treatment did not influence HBV DNA levels in blood, HBsAg levels in liver, and HBeAg in blood. An additive inhibitory effect was observed when CRV431 was combined with a reverse transcriptase inhibitor the nucleotide analog tenofovir exalidex (TXL) (formerly known as CMX157) [35]. Like CRV431, TXL decreased liver HBV DNA levels, but did not modify HBV DNA levels in blood, HBsAg levels in liver, and HBeAg in blood. Similar to other nucleos(t)ide drugs, TXL also did not reduce HBsAg in serum. No apparent toxicity was detected in drug-treated mice. Our study reveals the possibility of the use of CRV431 as partner in new anti-HBV drug regimens by showing for the first time that the host-targeting agent CRV431 alone or in combination with a direct-acting agent (TXL), significantly decreased liver HBV levels and could reduce HBsAg in serum.

## Materials and methods

### Drugs

CRV431 was synthesized in-house by chemical modification of cyclosporin A. Its purity exceeded 95% as determined by HPLC. TXL—a modified tenofovir drug with enhanced liver uptake—was manufactured by Cambrex High Point Inc. (NC, USA) to a purity of >98% as determined by HPLC.

### CRV431 serum concentration quantification

CRV431 concentrations in serum were determined from samples collected 3 hours following the final dose (Day 16). CRV431 was quantified using an on-line separation liquid chromatographic-electrospray ionization-mass spectrometry (O-SPE-LC-ESI-MS) method. Analytical runs consisted of serum standards, serum quality controls and study samples, which included cyclosporine A as the internal standard. Standards, controls and study samples were extracted using a zinc sulfate precipitation method. The supernatant from the precipitation procedure was transferred to autosampler vials for analysis. A Zorbax SB C18 Rapid Resolution HT analytical column (4.6 x 30 mm, 1.8 μm) with a Luna C8 guard column (2 x 20 mm) and a Zorbax SB-C8 capture column (2.1 mm x 12.5 mm, 5 μm) were used for chromatographic separation. The following ions (sodium adducts) were monitored: 1224.8 mass units for CsA and 1326.8 mass units for CRV431. The volume of serum used for analysis was 50 μl, and the measured concentration range was from the lower limit of quantitation (LLOQ) of 0.1 μg/mL to the upper limit of quantitation (ULOQ) of 10 μg/mL with a correlation coefficient (r^2^) of at least 0.998.

### HBV transgenic mouse study

Female transgenic mice HBV transgenic Tg05 mice (C57BL/6) provided by Dr. J. Ou [36] were pre-screened for serum levels of HBV DNA, HBsAg, and HBeAg, and high expressers were block-randomized into 6 groups. The criteria used to determine whether HBV transgenic mice were selected as high expressers were i) number of HBV DNA copies per mL of serum quantified by qPCR higher than 70; ii) ng of HBsAg per mL of serum quantified by ELISA higher than 700; and iii) ng of HBeAg per mL of serum quantified by ELISA higher than 700. CRV431 was dissolved in polyethylene glycol 300 molecular weight (PEG-300) and administered once-daily by oral gavage at 10 or 50 mg/kg/day for 16 days. PEG-300 vehicle was dosed at equivalent volumes. TXL was dissolved in phosphate-buffered saline and administered once-daily by oral gavage at 5 or 10 mg/kg/day for 16 days. Moreover, a combination of CRV431 (low dose of 10 mg/kg) and TXL (low dose of 5 mg/kg) was administered once-daily by oral gavage for 16 days. Liver, kidney and blood collection as well as necropsies were performed after 16 days of dosing. Liver DNA was isolated and analyzed by qPCR as previously described [33]. Serum and liver HBeAg and HBsAg were quantified by enzyme-linked immunosorbent assays (International Immuno Diagnostics). For liver HBsAg quantification, pieces of liver were homogenized by sonication in protein extraction buffer (10 mM Tris-HCl, 1% NP-40, 2.5 M NaCl, 1 mM EDTA, 0.04% NaN3, and1 mM PMSF at pH 7.5) with freshly added 1% protease inhibitors, and centrifuged at 27,000g for 20 min. Supernatants were collected and protein concentration determined by Pierce Protein Reagent Assay BCA Kit and levels of HBsAg quantified by enzyme-linked immunosorbent assay. Mouse weights were measured at 6-time points throughout the study.

For tissue DNA and RNA analyses, frozen liver (left lobe) and kidney tissues were mechanically pulverized under liquid nitrogen, and total genomic DNA and RNA were isolated for Southern and Northern blot analyses as previously described [37]. Membranes were analyzed for HBV DNA, HBV RNA and glyceraldehyde-3-phosphate dehydrogenase (GAPDH) as previously described [38].

Transgenic mice were maintained at the Department of Animal Resources (DAR) at TSRI in accordance with protocols approved by the TSRI Ethics Committee, the Institutional Animal Care and Use Committee (Protocol Number: 11–0015). This study was carried out in strict accordance with the recommendations in the Guide for the Care and Use of Laboratory Animals of the National Institutes of Health. All efforts were made to minimize suffering. The method of sacrifice used for the experimental mice was cervical dislocation. A power calculation was used to determine the sample size (number of mice/group).

### Statistical analyses

A paired t-test analysis was conducted between the group of vehicle-treated mice and each group of drug (CRV431 or TXL)-treated mice.

## Results

**Fig 1:**
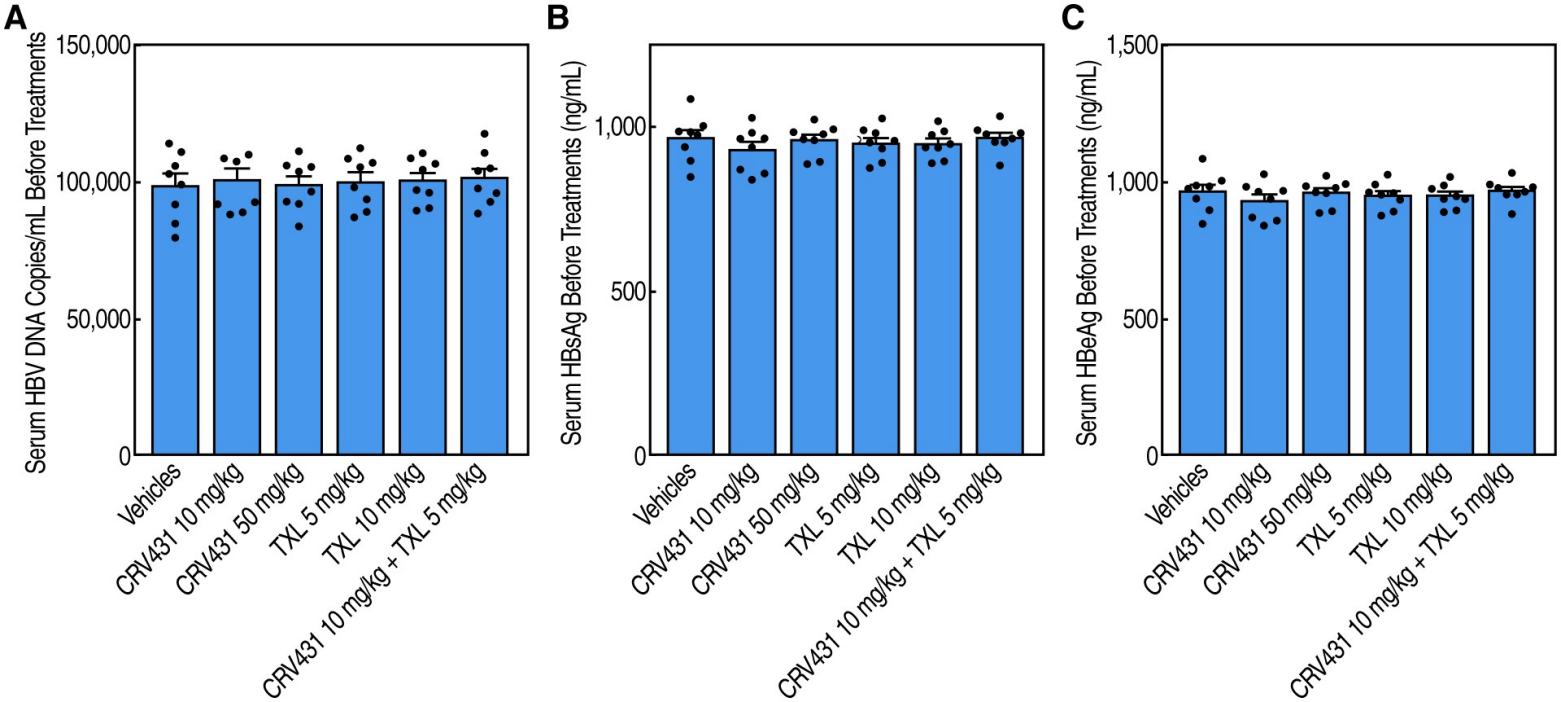
Pre-screening of HBV transgenic mice. HBV Tg05 mice (n = 48) were selected for their high serum levels in HBV DNA (**A**), HBsAg (**B**) and HBeAg (**C**).

HBV transgenic mice contain a complete viral genome integrated into the chromosomes that is constitutively transcribed at high levels in hepatic and renal cells [37]. HBV transgenic mice were pre-screened for serum levels of HBV DNA (Fig 1A), HBsAg (Fig 1B) and HBeAg (Fig 1C) levels to select high-expressers (S1 Table), and block-randomized into groups of 8 mice. Mice were treated daily for a period of 16 days with i) vehicle; ii) 10 mg/kg of CRV431; iii) 50 mg/kg of CRV431; iv) 5 mg/kg of TXL; v) 10 mg/kg of TXL; and a combination of 10 mg/kg of CRV431 and 5 mg/kg of TXL.

**Fig 2:**
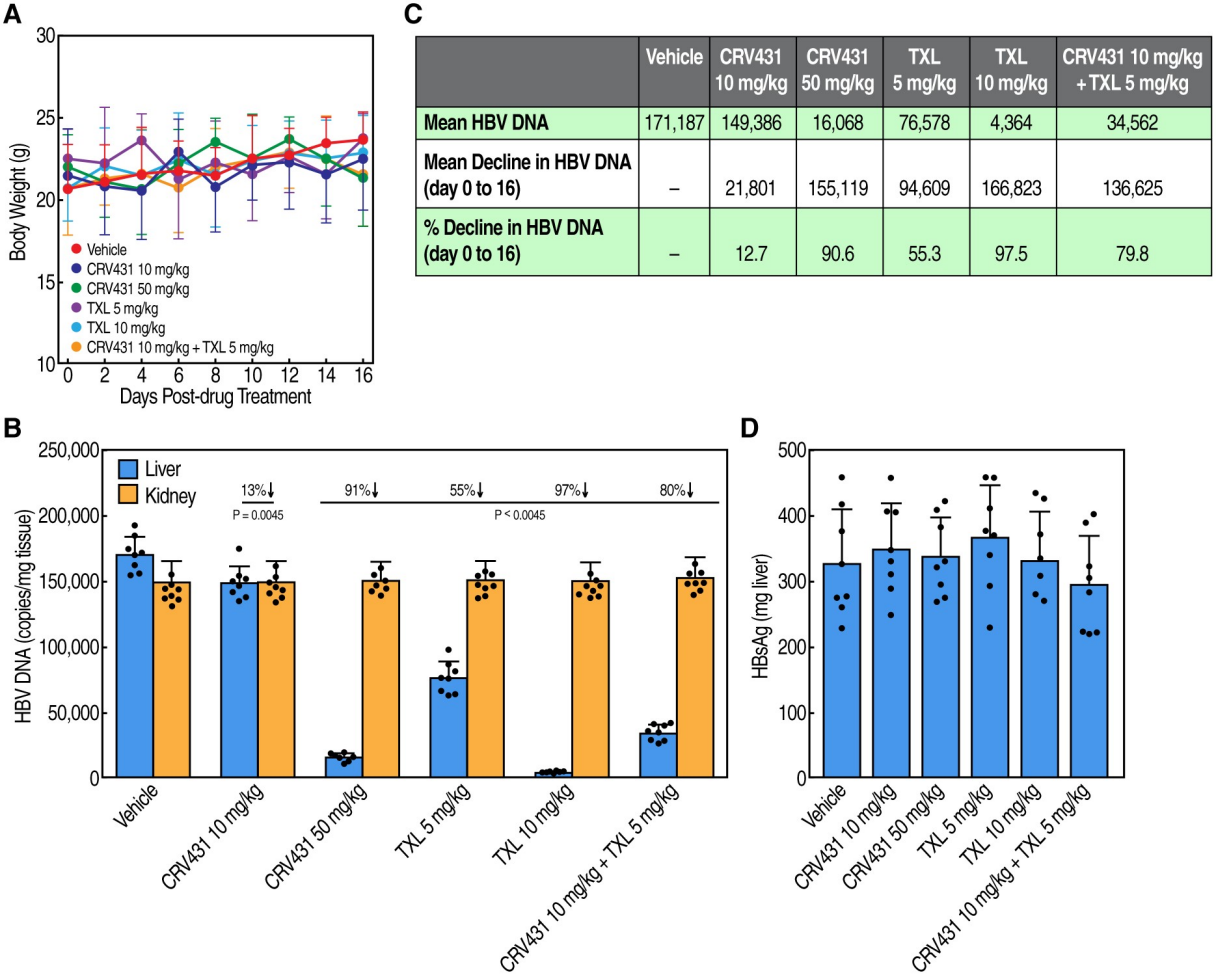
Body weight analysis and liver, kidney and serum HBV marker analysis. Daily body weight analysis of HBV transgenic mice treated with vehicle, CRV431, TXL and a combination of CRV431 and TXL for a period of 16 days (**A**). Liver and kidney HBV DNA (**B** and **C**) and liver HBsAg (**D**) analyses in HBV transgenic mice after 16-day treatments with vehicle, CRV431 TXL and a combination of CRV431 and TXL.

**Fig 3:**
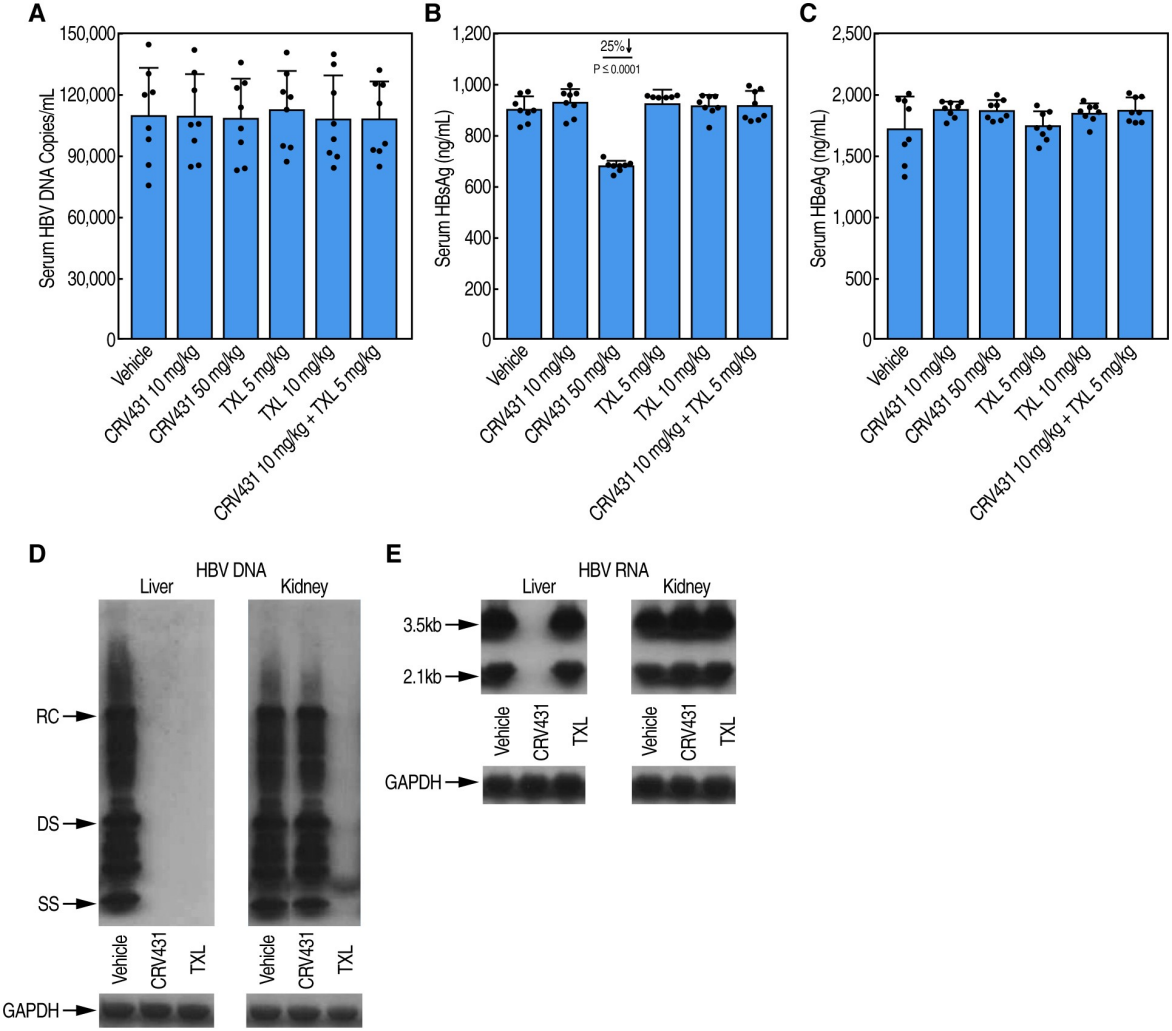
HBV marker analysis. HBV DNA (**A**), HBsAg (**B**) and HBeAg (**C**) serum analysis in HBV transgenic mice after 16-day treatments with vehicle, CRV431 TXL and a combination of CRV431 and TXL. Southern (**D**) and Northern (**E**) blot analyses for HBV DNA, HBV RNA and GAPDH in liver and kidney.

We found no overt signs of toxicity nor differences in mouse body weights among the vehicle and drug treatment groups over the 16-day period treatment (Fig 2A) (S2 Table). Liver HBV DNA levels were decreased both by CRV431 and TXL (Fig 2B and 2C) (S2 Table). While the low dose of CRV431 (10 mg/kg/day) reduced HBV DNA levels by only 13% compared to the vehicle group, the higher dose (50 mg/kg) reduced the mean HBV DNA level by 91%. Serum CRV431 drug levels at 3 hr post-dose on the final day of dosing were 0.01 μg/ml (± 0.01 μg/ml SD) and 0.34 μg/ml (0.22 μg/ml SD) in the low-dose and high-dose groups, respectively. TXL, a pro-drug of the nucleotide analog, tenofovir, also decreased liver HBV DNA levels dose-dependently. The reductions in HBV DNA were 55% and 97% for low-dose TXL (5 mg/kg/day) and high-dose TXL (10 mg/kg/day), respectively. In mice that were co-dosed with low doses of CRV431 (10 mg/kg/day) and TXL (5 mg/kg/day), liver HBV DNA decreased by 80% compared to the vehicle group. No reductions in liver HBsAg (Fig 2D) (S2 Table), serum HBV DNA (Fig 3A), or serum HBeAg (Fig 3C) were observed among any drug treatment. The lack of HBV DNA reduction in kidneys of HBV transgenic mice (Fig 2B) may explain the lack of serum HBV DNA reduction (Fig 3A) (S3 Table). However, there was a 25% reduction in HBsAg levels in the serum of 50 mg/kg/day CRV431-treated mice (Fig 3B) (S3 Table) but not in the liver (Fig 2D). The decline in the serum is unlikely to be a consequence of the reduction in the liver HBV DNA because there is no change in liver HBsAg (Fig 2D), and HBV transgenic mice do not make the HBV cccDNA transcriptional template. Therefore, the HBV DNA replication forms that decline in the liver upon treatment are likely encapsidated single-stranded DNA (SS) and relaxed circular form (RC) forms that would not be a source of HBsAg mRNA expression. Thus, we examined by Southern and Northern blot analyses, HBV DNA and RNA levels in liver and kidney of vehicle-, CRV431- and TXL-treated mice. We found that the reduction in liver HBV DNA by CRV431 analyzed by Southern blot (Fig 3D) correlate with that analyzed by qPCR (Fig 2B). No HBV DNA reduction by CRV431 was observed in kidney (Fig 3D). Similar HBV RNA reduction was observed in the kidney of CRV431-treated mice (Fig 3E). The reverse transcriptase inhibitor TXL reduced HBV DNA levels in both liver and kidney but did not reduce HBV RNA levels in both liver and kidney (Fig 3E).

## Discussion

Current therapies for HBV treatment include interferons (IFN)s and nucleos(t)ide analogs. IFN treatments are associated with side-effects, and antiviral treatments do not lead to the elimination of the virus due to the persistence of cccDNA in HBV-infected hepatocytes. Therefore, there is a critical need for the development of improved therapies for HBV patients.

In this study, we examined the potential inhibitory effect of the cyclophilin inhibitor—CRV431—in HBV transgenic mice. For the first time, we found that a 16-day daily administration of CRV431 greatly reduced HBV DNA in the liver of transgenic mice. The inhibition was dose-dependent. The mechanisms of inhibitory action of CRV431 remain unknown. A previous study demonstrated an interaction between CypA and HBsAg and suggested that the CypA-HBsAg complex formation may play an important role in HBV pathogenesis [39]. A more recent study confirmed a direct contact between HBsAg and CypA, and that CRV431 prevented the interaction between the viral and the host proteins [40]. However, to date, no demonstration of a relationship between CypA-HBsAg complex formation and HBV DNA replication has yet been provided. Interestingly, another recent study presented evidence that CypA binds to another HBV protein—HBx, and that similarly to CypA-HBsAg interactions, CRV431 prevented CypA-HBx complex formation in a dose-dependent manner [41]. Recently it has been reported that two members of the parvulin class of peptidyl-prolyl *cis-trans* isomerases, which are closely related to cyclophilins, also bind to HBx as well as cccDNA and regulate HBV transcription and replication [42]. As for CypA-HBsAg interactions, no mechanistic link between CypA-HBx complex formation and HBV DNA replication has yet been provided or demonstrated. In other words, even if these interactions exist, their relevance for HBV replication and/or pathogenesis remains to be demonstrated. Therefore, further work is required to elucidate the mechanisms of action that lead to an inhibition of HBV DNA replication in the liver of transgenic mice treated with the cyclophilin inhibitor CRV431.

It is important to emphasize that the HBV transgenic mouse model does not fully recapitulate the HBV life cycle. HBV transgenic mice lack: i) human NTCP, the receptor necessary for HBV entry into cells; ii) cccDNA formation; and iii) viral spread [43]. This suggests that the CRV431 inhibition of liver HBV DNA in transgenic mice is not mediated by either a block of NTCP entry and/or the elimination of cccDNA. Another issue with this model is that transgenic mice contain a complete viral genome integrated into the chromosomes that is transcribed at high levels not only in hepatic cells, but also in renal cells [37]. If the CRV431-mediated HBV DNA block effect is specific for hepatocytes, only a partial or no inhibitory effect would be observed in total HBV DNA levels in serum. This could explain the lack of effect of CRV431 on HBV DNA and HBeAg levels in serum of drug-treated transgenic mice. Further work will be required to understand the exclusive inhibition of HBV DNA replication in the liver of transgenic mice.

The modest, but statistically significant reduction in HBsAg levels in the serum of CRV431-treated mice (high dose) could be the consequence of the elimination of a fraction of HBsAg from the serum compartment but not the liver. Since HBsAg binds CypA, one could speculate that they are bound together in serum and that HBsAg is cleared more slowly when bound to CypA. Conversely, by reducing CypA-HBsAg binding, CRV431 may elevate the clearance rate of HBsAg. Because the levels of liver HBsAg do not change, the decrease in serum HBsAg could be due to an inhibition of HBsAg secretion. However, the observable HBsAg reduction in the serum means that unlike HBV DNA inhibition, this inhibition would have to occur in both the liver and kidney. If there is an inhibition of HBsAg secretion from the liver, it should be minimal since we do not observe higher HBsAg levels in the liver of CRV431-treated mice.

The reduction in HBV DNA levels in the liver of transgenic mice suggests that CRV431 interfered with HBV DNA replication in murine hepatocytes. Our observation that the combination of low doses of CRV431 and the nucleotide analog TXL exhibited an additive effect in the inhibition of liver HBV DNA levels might be due to the two compounds acting on the same step in the HBV life cycle. TXL exerts its therapeutic actions by inhibiting HBV polymerase-mediated HBV DNA elongation, but there is no known binding of cyclophilins to HBV polymerase nor participation of cyclophilins in DNA elongation. The combinational effect of CRV431 (host-targeting) and TXL (direct-acting) on HBV DNA production is more consistent with the two compounds acting on distinct steps of the HBV life cycle.

To date, non-immunosuppressive cyclosporine A analogs have been shown to be safe in humans. For example, phase I, II, and III studies in HCV patients demonstrated the safety of oral daily high doses of the prototypical cyclosporine A analog alisporivir [15–18] as well as other cyclosporine A analogs such as NIM811 [44] and SCY-635 [45]. This is in accordance with the fact that CypA-knockout mice or human T cells are viable and healthy [46–47]. These findings minimize the risk of the development of CRV431 as a new anti-HBV agent. Our demonstration that CRV431 significantly reduced liver HBV DNA levels and slightly reduced serum HBsAg levels indicates that this cyclosporine A analog has the potential to serve as drug partner in new and improved HBV regimens.

## Supporting information

S1 Table Data for Fig 1: Pre-screening of HBV DNA, HBsAg and HBeAg serum levels in HBV transgenic mice before drug treatments. Data for serum HBV DNA (copies/mL), HBsAg and HBeAg (ng/mL) levels are expressed as mean of triplicate with standard deviations (SD).
(XLSX)

S2 Table **Data for** Fig 2A**: Body weight of mice**. Data of body weight (g) of mice measured every two day for a period of 16 days are expressed as mean of triplicate with standard deviations (SD). **Data for** Fig 2B**: Amounts of HBV DNA in kidney and liver of mice**. Data are expressed in numbers of HBV DNA copies per mg of kidney or liver tissue. Data are expressed as mean of triplicate with standard deviations (SD). **Data for** Fig 2D**: Amounts of HBsAg in liver of mice**. Data are expressed in pg of HBsAg per mg of liver tissue. Data are expressed as mean of triplicate with standard deviations (SD).
(XLSX)

S3 Table Data for Fig 3: Levels of HBV DNA, HBsAg and HBeAg in serum of mice after drug treatments. Data for serum HBV DNA (copies/mL), HBsAg and HBeAg (ng/mL) levels are expressed as mean of triplicate with standard deviations (SD).
(XLSX)

## References

[1] Liu CJ, Chen PJ (2014) Updates on the treatment and outcomes of dual chronic hepatitis C and B virus infection. World J Gastroenterol 20:2955–2961. 10.3748/wjg.v20.i11.2955 24659886 PMC3961993

[2] Ott JJ, Stevens GA, Groeger J, Wiersma ST. Global epidemiology of hepatitis B virus infection: new estimates of age-specific HBsAg seroprevalence and endemicity. Vaccine 2012;30:2212–2219. 10.1016/j.vaccine.2011.12.116 22273662

[3] Liu CY, Chen KF, Chen PJ (2015) Treatment of liver cancer. Cold Spring Harb Perspect Med 5:289–304. 10.1101/cshperspect.a021535 PMC4561392 26187874

[4] Seeger C, Mason WS (2015) Molecular biology of hepatitis B virus infection. Virology 479–480:672–686. 10.1016/j.virol.2015.02.031 25759099 PMC4424072

[5] Zeisel MB, Lucifora J, Mason WS, Sureau C, Beck J, Levrero M, et al (2015) Towards an HBV cure: state-of-the-art and unresolved questions-report of the ANRS workshop on HBV cure. Gut 64:1314–1326. 10.1136/gutjnl-2014-308943 25670809

[6] Boettler T, Moradpour D, Thimme R, Zoulim F (2014) Bridging basic science and clinical research: the EASL Monothematic Conference on Translational Research in Viral Hepatitis. J Hepatol 61:696–705. 10.1016/j.jhep.2014.05.016 24845610

[7] Brahmania M, Feld J, Arif A, Janssen HL (2016) New therapeutic agents for chronic hepatitis B. Lancet Infect Dis 16:e10–e21. 10.1016/S1473-3099(15)00436-3 26795693

[8] Pawlotsky JM, Dusheiko G, Hatzakis A, Lau D, Lau G, Liang TJ, et al (2008) Virologic monitoring of hepatitis B virus therapy in clinical trials and practice: recommendations for a standardized approach. Gastroenterology 134:405–415. 10.1053/j.gastro.2007.11.036 18242209 PMC2676233

[9] Feeney ER, Chung RT (2014) Antiviral treatment of hepatitis C. BMJ 348:g3308 10.1136/bmj.g3308 25002352 PMC6880951

[10] Lindenbach BD, Rice CM (2013) The ins and outs of hepatitis C virus entry and assembly. Nat Rev Microbiol 11:688–700. 10.1038/nrmicro3098 24018384 PMC3897199

[11] Hopkins S, Gallay PA (2015) The role of immunophilins in viral infection. Biochim Biophys Acta 1850:2103–2110. 10.1016/j.bbagen.2014.11.011 Epub 2014 Nov 18. 25445708 PMC4491039

[12] Gallay PA, Bobardt MD, Chatterji U, Trepanier DJ, Ure D, Ordonez C, et al (2015) The Novel Cyclophilin Inhibitor CPI-431-32 Concurrently Blocks HCV and HIV-1 Infections via a Similar Mechanism of Action. PLoS One. 2015 8 11;10(8):e0134707 10.1371/journal.pone.0134707 eCollection 2015. 26263487 PMC4532424

[13] de Wilde AH, Pham U, Posthuma CC, Snijder EJ. (2018) Cyclophilins and cyclophilin inhibitors in nidovirus replication. Virology 522:46–55. 10.1016/j.virol.2018.06.011 30014857 PMC7112023

[14] Chatterji U, Garcia-Rivera JA, Baugh J, Gawlik K, Wong KA, Zhong W, et al (2014) The combination of alisporivir plus an NS5A inhibitor provides additive to synergistic anti-hepatitis C virus activity without detectable cross-resistance. Antimicrob Agents Chemother 58:3327–3334. 10.1128/AAC.00016-14 Epub 2014 Mar 31. 24687498 PMC4068438

[15] Pawlotsky JM, Flisiak R, Sarin SK, Rasenack J, Piratvisuth T, Chuang WL, et al (2015) Alisporivir plus ribavirin, interferon free or in combination with pegylated interferon, for hepatitis C virus genotype 2 or 3 infection. Hepatology 62:1013–23. 10.1002/hep.27960 26118427

[16] Zeuzem S, Flisiak R, Vierling JM, Mazur W, Mazzella G, Thongsawat S, et al (2015) Randomised clinical trial: alisporivir combined with peginterferon and ribavirin in treatment-naïve patients with chronic HCV genotype 1 infection (ESSENTIAL II). Aliment Pharmacol Ther. 2015 10;42(7):829–44. 10.1111/apt.13342 Epub 2015 Aug 4. 26238707

[17] Pawlotsky JM, Flisiak R, Sarin SK, Rasenack J, Piratvisuth T, Chuang WL, et al (2015) Alisporivir plus ribavirin, interferon free or in combination with pegylated interferon, for hepatitis C virus genotype 2 or 3 infection. Hepatology 62:1013–23. 10.1002/hep.27960 Epub 2015 Aug 10. 26118427

[18] Buti M, Flisiak R, Kao JH, Chuang WL, Streinu-Cercel A, Tabak F, et al (2015) Alisporivir with peginterferon/ribavirin in patients with chronic hepatitis C genotype 1 infection who failed to respond to or relapsed after prior interferon-based therapy: FUNDAMENTAL, a Phase II trial. J Viral Hepat 22:596–606. 10.1111/jvh.12360 Epub 2014 Nov 21. 25412795

[19] Lin K, Gallay P. (2013) Curing a viral infection by targeting the host: the example of cyclophilin inhibitors. Antiviral Res 99:68–77. 10.1016/j.antiviral.2013.03.020 Epub 2013 Apr 8. 23578729 PMC4332838

[20] Gallay PA, Lin K. (2013) Profile of alisporivir and its potential in the treatment of hepatitis C. Drug Des Devel Ther. 2013;7:105–15. 10.2147/DDDT.S30946 Epub 2013 Feb 15. 23440335 PMC3578503

[21] Hopkins S, Gallay P. (2012) Cyclophilin inhibitors: an emerging class of therapeutics for the treatment of chronic hepatitis C infection. Viruses 4:2558–77. 10.3390/v4112558 23202494 PMC3509662

[22] Baugh JM, Garcia-Rivera JA, Gallay PA (2013) Host-targeting agents in the treatment of hepatitis C: a beginning and an end? Antiviral Res 100:555–61. 10.1016/j.antiviral.2013.09.020 Epub 2013 Sep 30. 24091203 PMC3971122

[23] Franke EK, Yuan HE, Luban J. (1994) Specific incorporation of cyclophilin A into HIV-1 virions. Nature 372:359–62. 10.1038/372359a0 7969494

[24] Thali M, Bukovsky A, Kondo E, Rosenwirth B, Walsh CT, Sodroski J, et al (1994) Functional association of cyclophilin A with HIV-1 virions. Nature 372:363–365. 10.1038/372363a0 7969495

[25] Rosenwirth B, Billich A, Datema R, Donatsch P, Hammerschmid F, Harrison R, et al (1994) Inhibition of human immunodeficiency virus type 1 replication by SDZ NIM 811, a nonimmunosuppressive cyclosporine analog. Antimicrob Agents Chemother 38:1763–72. 10.1128/aac.38.8.1763 7527198 PMC284634

[26] Hanoulle X, Badillo A, Wieruszeski JM, Verdegem D, Landrieu I, Bartenschlager R, et al (2009) Hepatitis C virus NS5A protein is a substrate for the peptidyl-prolyl cis/trans isomerase activity of cyclophilins A and B. J Biol Chem 284:13589–601. 10.1074/jbc.M809244200 Epub 2009 Mar 18. 19297321 PMC2679460

[27] Coelmont L, Hanoulle X, Chatterji U, Berger C, Snoeck J, Bobardt M, et al (2010) DEB025 (Alisporivir) inhibits hepatitis C virus replication by preventing a cyclophilin A induced cis-trans isomerisation in domain II of NS5A. PLoS One 5:e13687 10.1371/journal.pone.0013687 21060866 PMC2965138

[28] Chatterji U, Lim P, Bobardt MD, Wieland S, Cordek DG, Vuagniaux G, et al (2010) HCV resistance to cyclosporin A does not correlate with a resistance of the NS5A-cyclophilin A interaction to cyclophilin inhibitors. J Hepatol 53:50–6. 10.1016/j.jhep.2010.01.041 Epub 2010 Apr 3. 20451281 PMC2884070

[29] Fernandes F, Ansari IU, Striker R. (2010) Cyclosporine inhibits a direct interaction between cyclophilins and hepatitis C NS5A. PLoS One 9:e9815 10.1371/journal.pone.0009815 20352119 PMC2843741

[30] Shimura S, Watashi K, Fukano K, Peel M, Sluder A, Kawai F, et al (2017) Cyclosporin derivatives inhibit hepatitis B virus entry without interfering with NTCP transporter activity. J Hepatol 66:685–692. 10.1016/j.jhep.2016.11.009 Epub 2016 Nov 25. 27890789 PMC7172969

[31] Watashi K, Sluder A, Daito T, Matsunaga S, Ryo A, Nagamori S, et al (2014) Cyclosporin A and its analogs inhibit hepatitis B virus entry into cultured hepatocytes through targeting a membrane transporter, sodium taurocholate cotransporting polypeptide (NTCP). Hepatology 59:1726–37. 10.1002/hep.26982 Epub 2014 Apr 1. 24375637 PMC4265264

[32] Nkongolo S, Ni Y, Lempp FA, Kaufman C, Lindner T, Esser-Nobis K, et al (2014) Cyclosporin A inhibits hepatitis B and hepatitis D virus entry by cyclophilin-independent interference with the NTCP receptor. J Hepatol 60:723–31. 10.1016/j.jhep.2013.11.022 Epub 2013 Dec 1. 24295872

[33] Phillips S, Chokshi S, Chatterji U, Riva A, Bobardt M, Williams R, et al (2015) Alisporivir inhibition of hepatocyte cyclophilins reduces HBV replication and hepatitis B surface antigen production. Gastroenterology 148:403–14.e7. 10.1053/j.gastro.2014.10.004 Epub 2014 Oct 8. 25305505 PMC7172380

[34] Hansson MJ, Moss SJ, Bobardt M, Chatterji U, Coates N, Garcia-Rivera JA, et al (2015) Bioengineering and semisynthesis of an optimized cyclophilin inhibitor for treatment of chronic viral infection. Chem Biol 22:285–92. 10.1016/j.chembiol.2014.10.023 Epub 2015 Jan 22. 25619934 PMC4336584

[35] Painter GR, Almond MR, Trost LC, Lampert BM, Neyts J, De Clercq E, et al (2007) Evaluation of hexadecyloxypropyl-9-R-[2-(Phosphonomethoxy)propyl]- adenine, CMX157, as a potential treatment for human immunodeficiency virus type 1 and hepatitis B virus infections. Antimicrob Agents Chemother 51:3505–9. Epub 2007 Jul 23. 10.1128/AAC.00460-07 17646420 PMC2043283

[36] Zheng Y, Chen WL, Louie SG, Yen TS, Ou JH. (2007) Hepatitis B virus promotes hepatocarcinogenesis in transgenic mice. Hepatology 45:16–21. 10.1002/hep.21445 17187428

[37] Guidotti LG, Matzke B, Schaller H, Chisari FV (1995) High-level hepatitis B virus replication in transgenic mice. J Virol 69: 6158–6169. 7666518 10.1128/jvi.69.10.6158-6169.1995 PMC189513

[38] Guidotti LG, Borrow P, Hobbs MV, Matzke B, Gresser I, Oldstone MB, et al Viral cross talk: intracellular inactivation of the hepatitis B virus during an unrelated viral infection of the liver. Proc Natl Acad Sci U S A. 1996 5 14;93(10):4589–94. 10.1073/pnas.93.10.4589 8643448 PMC39321

[39] Tian X, Zhao C, Zhu H, She W, Zhang J, Liu J, et al (2010) Hepatitis B Virus (HBV) Surface Antigen Interacts with and Promotes Cyclophilin A Secretion: Possible Link to Pathogenesis of HBV Infection J Virol 84: 3373–3381. Published online 2010 Jan 20. 10.1128/JVI.02555-09 20089655 PMC2838095

[40] Foster R, Ure D, Trepanier D, Sullivan-Bolyai J, Matkovits T, Greytok J, et al. (2017) CRV431: Multiple Therapeutic Actions In Vitro and In Vivo. HEP DART 2017, Kona, Hawaii.

[41] Foster R, Ure D, Trepanier D, Gallay P (2017). The cyclophilin inhibitor CRV431 prevents both HBx-cyclophilin complex formation and HBV replication. The International Liver Congress^™^ EASL 2017—European Association for the Study of the Liver Amsterdam, The Netherlands.

[42] Saeed U, Kim J, Piracha ZZ, Kwon H, Jung J, Chwae YJ, et al (2018) Parvulin 14 and parvulin 17 bind to HBx and cccDNA and upregulate HBV replication from cccDNA to virion in a HBx-dependent manner. J Virol. 2018 12 19. [Epub ahead of print] 10.1128/JVI.01840-18 PMC6401437 30567987

[43] Hwang JR, Park SG (2018) Mouse models for hepatitis B virus research. Lab Anim Res 34:85–91. 10.5625/lar.2018.34.3.85 Epub 2018 Sep 27. 30310404 PMC6170223

[44] Lawitz E, Godofsky E, Rouzier R, Marbury T, Nguyen T, Ke J, et al (2011) Safety, pharmacokinetics, and antiviral activity of the cyclophilin inhibitor NIM811 alone or in combination with pegylated interferon in HCV-infected patients receiving 14 days of therapy. Antiviral Res 89(3):238–45. 10.1016/j.antiviral.2011.01.003 Epub 2011 Jan 19. 21255610

[45] Hopkins S, DiMassimo B, Rusnak P, Heuman D, Lalezari J, Sluder A, et al (2012) The cyclophilin inhibitor SCY-635 suppresses viral replication and induces endogenous interferons in patients with chronic HCV genotype 1 infection. J Hepatol 57(1):47–54. 10.1016/j.jhep.2012.02.024 Epub 2012 Mar 13. 22425702

[46] Colgan J, Asmal M, Luban J (2000) Isolation, characterization and targeted disruption of mouse ppia: cyclophilin A is not essential for mammalian cell viability. Genomics 68(2):167–78. 10.1006/geno.2000.6295 10964515

[47] Braaten D, Luban J (2001) Cyclophilin A regulates HIV-1 infectivity, as demonstrated by gene targeting in human T cells. EMBO J 20(6):1300–9. 10.1093/emboj/20.6.1300 11250896 PMC145517

